# Correlates of intimate partner violence among pregnant and parenting adolescents: a cross-sectional household survey in Blantyre District, Malawi

**DOI:** 10.1186/s12978-023-01606-y

**Published:** 2023-04-13

**Authors:** Juliet Amarachukwu Nwafor, Elita Chamdimba, Anthony Idowu Ajayi, Boniface Ayanbekongshie Ushie, Alister C. Munthali, Chrissie Thakwalakwa, Caroline W. Kabiru

**Affiliations:** 1 Sexual, Reproductive, Maternal, New-Born, Child, and Adolescent Health (SRMNCAH) Unit, African Population and Health Research Center, Manga Close, Nairobi, 00100 Kenya; 2 Centre for Social Research, University of Malawi, P. O. Box 280, Zomba, Malawi

**Keywords:** Intimate partner violence, Domestic violence, Pregnant, Parenting, Adolescents, Malawi

## Abstract

**Background:**

Despite efforts from the government and developmental partners to eliminate gender-based violence, intimate partner violence (IPV) remains a pervasive global health and human rights problem, affecting up to 753 million women and girls globally. Few studies on IPV have focused on pregnant and parenting adolescent (PPA) girls in Africa, although the region has the highest rates of adolescent childbearing. This limited attention results in the neglect of pregnant and parenting adolescents in policies and interventions addressing IPV in the region. Our study examined IPV prevalence and its individual, household, and community-level correlates among pregnant and parenting adolescent girls (10–19 years) in Blantyre District, Malawi.

**Methods:**

We collected data from a cross-section of pregnant and parenting adolescent girls (n = 669) between March and May 2021. The girls responded to questions on socio-demographic and household characteristics, lifetime experience of IPV (i.e., sexual, physical, and emotional violence), and community-level safety nets. We used multilevel mixed-effect logistic regression models to examine the individual, household, and community-level factors associated with IPV.

**Results:**

The lifetime prevalence of IPV was 39.7% (n = 266), with more girls reporting emotional (28.8%) than physical (22.2%) and sexual (17.4%) violence. At the individual level, girls with secondary education (AOR: 1.72; 95% CI: 1.16–2.54), who engaged in transactional sex (AOR: 2.29; 95% CI: 1.35–3.89), and accepted wife-beating (AOR: 1.97; 95% CI: 1.27–3.08) were significantly more likely to experience IPV compared to those with no education/primary education, who never engaged in transactional sex and rejected wife beating. Girls aged 19 (AOR: 0.49; 95% CI: 0.27–0.87) were less likely to report IPV than those aged 13–16. At the household level, girls with fair and poor partner support had higher odds of experiencing IPV, but the effect size did not reach a significant level in the parsimonious model. A high perception of neighborhood safety was associated with a lower likelihood of experiencing IPV (AOR: 0.81; 95% CI: 0.69–0.95).

**Conclusion:**

Intimate partner violence is rife among pregnant and parenting adolescent girls in Malawi, underscoring the need for appropriate interventions to curb the scourge. Interventions addressing IPV need to target younger adolescents, those engaging in transactional sex, and those having weaker community-level safety nets. Interventions to change social norms that drive the acceptance of gender-based violence are also warranted.

## Introduction

Despite efforts from the government and developmental partners to eliminate gender-based violence, intimate partner violence (IPV) remains a pervasive global health and human rights problem, affecting up to 753 million women and girls globally [1]. Women in Africa are disproportionately affected, with the prevalence of IPV at 33%, higher than in other regions, except Southern Asia (35%). However, there are variations across Africa. For example, 47% and 46% of women in the Democratic Republic of Congo and Equatorial Guinea, respectively, reported having ever experienced IPV compared to 24% in South Africa, Ghana, and Nigeria [1].

Intimate partner violence is a global concern because it has many short and long-term adverse health and socioeconomic consequences for the victims, their families, and society [2, 3]. For example, women who experience IPV are more likely to report depression, anxiety, low self-esteem, and suicide or attempted suicide [4]. Further, IPV survivors experiencing poor mental and physical health are more likely to be less productive, thus reducing their contributions to societal development [3, 5]. In addition, studies have linked IPV to poor sexual and reproductive health (SRH) and child health outcomes, including HIV, unintended pregnancies, pregnancy loss, suboptimal breastfeeding, and infant and child mortality [6, 7].

Although studies generally focus on women and girls of reproductive age (15–49 years), the literature suggests a preponderance of IPV among adolescent girls (10–19 years) [8, 9]. Intimate partner violence starts early, with approximately one in four girls aged 15–19 years already subjected to physical or sexual violence [1]. The effects of violence on their health and socioeconomic well-being are damaging and lifelong. Exposure to sexual violence during adolescence places the survivors on a lifelong trajectory of violence [5, 10]. Abused and violated boys are more likely to become perpetrators themselves [11]. Girls who experienced abuse in their childhood faced increased and disproportionate levels of IPV throughout their life. The resulting poor mental health from childhood abuse continues into adulthood and hampers their productivity.

Existing research suggests that factors influencing IPV operate at multiple levels, including individual, household, and community [12]. For example, studies have shown that low educational attainment, low income, early marriage, and endorsing wife-beating are individual-level factors that increase women’s IPV risk [13, 14]. Partners’ alcohol use, controlling behavior, and childhood exposure to violence are household-level factors linked with IPV [9, 15]. Community-level drivers of IPV include unequal gender norms, rural residence, and unsafe neighborhoods.

Studies suggest varying IPV experiences among sub-categories of adolescents, with those living with HIV and disabilities disproportionately affected [16–18]. Despite the massive burden of adolescent childbearing in the African region, most studies exploring the experiences of IPV and interventions to combat it among pregnant and parenting adolescent (PPA) girls are in the Global North [19–23]. The lack of focus on IPV among PPAs in Africa has profound implications for neglecting the suffering young girls. Because of limited research on IPV among PPAs, the burden of the problem and factors driving exposure to violence among this cohort are not well understood, limiting the chances of decision-makers prioritizing adolescents for interventions.

While much research has been directed toward understanding the consequences of early and unintended pregnancy on girls’ physical and socioeconomic well-being, there is still limited attention to the lived experiences of the millions of girls who become pregnant in Africa yearly, including their exposure to IPV. Existing research, however, clearly shows that sexual violence contributes to early and unintended pregnancy among girls in the region [24]. Pregnant girls often consider marriage and cohabitation their best options [25]. Moreover, child marriage has many adverse effects on girls, including exposure to IPV [26]. Therefore, it is critical to draw decision-makers' attention to IPV among PPAs by researching their experiences, exposure risks, and protective factors. Our study fills this gap by examining the prevalence and individual, household, and community-level correlates of IPV among PPAs in Blantyre, Malawi.

Previous studies have shown that violence against women and girls is endemic in Malawi [8, 13, 15, 27]. According to the Violence Against Children and Young Women in Malawi Survey [28], 27% of females aged 13–24 years had experienced sexual violence once in their lifetime, and intimate partners or spouses perpetrated 31% of the cases, 28% by friends or neighbors. Over half of the victims of sexual violence told someone about it. Kidman and Kohler [15] studied ever-partnered adolescents aged 10–16 in Malawi and found 27% IPV prevalence, 24% among boys, and 31% among girls. Gender ideology was not significantly associated with IPV victimization, but the experience of childhood adversity reached a statistically significant level [15]. We found no study focusing on PPAs. Our study addresses this gap by examining IPV prevalence and its risk and protective factors among PPAs in the country.

### Theoretical underpinning

Intimate partner violence is a complex phenomenon with multiple-level determinants. We utilized the socio-ecological model as a theoretical framework to capture the nuanced factors—operating at multiple levels of social interactions—associated with IPV exposure among pregnant and parenting girls. This model has been applied in other studies with young people in Global South countries such as India [29] and Nigeria [30], among others. The socio-ecological model [31] holds that the ecology of development from childhood onwards happens at multiple levels made up of systems from the most immediate micro-level (e.g., interpersonal histories) to the larger macro-levels (e.g., society and institutions). At the micro level, a person’s beliefs, knowledge, and attitudes about gender norms, intimate relations, and cultural values inform their degree of IPV tolerance at the individual level.

Similarly, household interactions at the family level also play a role in determining the likelihood of IPV experiences because family members, friends, and intimate partners shape both the victims’ and perpetuators’ expectations, habits, and patterns related to sex, violence, respect, consent, and communication. This implies that for PPAs, the sense of empowerment that is expressed within intimate partnerships, is first developed and modeled at the family level. As childhood development progresses, the community level also becomes a key influence as a person is planted into their neighborhoods, schools, social groups, and jobs. Consequently, by drawing from the socio-ecological model, our study recognizes that characterizing IPV as a singularly represented phenomenon would be faulty. While we acknowledge that there are other larger systems beyond the community level, such as the macrosystems of society, it is important to note that our study has limited its application of the socio-ecological model to three levels—the individual, household, and community**.**

## Methods

### Study design

Data analyzed in this study were drawn from a cross-sectional survey of pregnant and parenting girls in rural and urban Blantyre District in southern Malawi. The larger study aimed to understand the lived experiences of PPAs aged 10–19 years. As of 2018, there were nearly 860,000 and 500,000 inhabitants in urban and rural Blantyre, respectively [32]. The rate of childbearing among adolescents aged 15–19 in Malawi in 2016 was 29% [33]. The 2015–16 Malawi Demographic and Health Survey showed that 19% of women in Malawi aged 25–49 had their first sexual intercourse before age 15 and 64% before age 18. The percentage of girls in Blantyre aged 15–19 who had already begun childbearing was 32.1%. Those in this age group who were pregnant with their first child was 4.4%; while 16.5% had already had a live birth [33].

### Sample size and selection of participants

A total sample of 669 PPAs of the 679 identified completed the survey; 10 refused to participate in the study. The sample size is sufficient to generate 80% statistical power for all the variables in the study. We based the sample estimation on the following parameters: in 2015, 29% of adolescent girls in Malawi had begun childbearing [33]; the proportion of adolescent fertility in the base population is 0.136 in Malawi; we considered a design effect of 1.5, a relative margin of error (RME) of 0.0325; average household size of five members in Malawi, and 5% possible incomplete responses.

We used a two-stage cluster random sampling to select study participants. In the first stage, we randomly selected 66 enumeration areas (EAs) from the Primary Sampling Frame developed by the Malawi National Statistical Office. Malawi is demarcated into small census clusters called EAs and stratified by urban and rural areas. In the second stage, we conducted a household listing in the selected clusters to identify all households with PPAs. We undertook the household listing to create an updated list of households for all selected EAs so that the sampled households represented the total population. All structures in randomly selected 66 EAs were listed, and households were identified. The listing exercise involved enumerating all household members and recording information on age, sex, and relationship to the household head.

Participants were included in the study if they were aged 10–19, ever pregnant (irrespective of the outcome of the pregnancy), currently pregnant, or had ever had a child regardless of their marital or relationship status, and provided consent to participate in the study. All PPAs identified in the households were eligible for the study. Interviews were conducted by well-trained and experienced research assistants using SurveyCTO installed on Android-powered tablets. The data collection took place between March and May 2021.

### Ethical consideration

The University of Malawi Research Ethics Committee (UNIMAREC) approved the study, and we observed all guidelines for conducting research with human participants. Research assistants were trained in research ethics before fieldwork. All participants provided voluntary informed consent after our team availed sufficient information about the study. Permission from parents and guardians was obtained for unmarried minors, while participants assented to participate. We anonymized all the data to protect participants' privacy and confidentiality.

### Variables and measurements

#### Dependent variable

The dependent variable was IPV. It was measured using 15 previously validated questions on sexual, physical, and emotional violence used in Demographic and Health Surveys. All PPAs reported whether their intimate partners had said something to humiliate, threaten to hurt or harm, or insult or make them feel bad. Eight questions assessed physical violence, focusing on whether intimate partners pushed, slapped, arm-twisted, hair-pulled, punched, beat up, choked, threatened with a knife, or attacked the respondent with a weapon. Four questions assessed sexual violence, encompassing grabbing or fondling, attempting to have sex against respondent's will, and physically forcing the respondent to have sex or perform sexual acts against their will. These questions combined, and any experience of one was considered IPV (yes coded as 1 and no coded as 0). The alpha coefficient for physical, emotional, and sexual violence were 0.83, 0.72, and 0.86, respectively, indicating high internal consistency among the items.

#### Independent variables

We included individual, household, and community variables based on the socio-ecological framework on violence against women. The socio-ecological model argues that factors that predispose women to violence operate on multiple levels, including individual, household, and community. Therefore, we considered age, marital status, employment, education, transactional sex, and endorsement of wife-beating at the individual level. Age was coded as 13–16, 17, 18, and 19. Marital status was categorized as single, married, and separated/divorced. Employment status was defined as working for pay or not, while education was categorized as no education, primary and secondary education. However, in the analysis, we combined the no education and primary categories because only one participant had no formal education of the 669 respondents.

We used nine questions to measure transactional sex. These questions touched on engaging in sex for money, food, shelter, school fees, phone/airtime, clothes/shoes/beauty products, sanitary pads, protection, and rent. All the questions demonstrated high internal consistency with a Cronbach alpha coefficient of 0.78. Engagement in sex for any items listed was considered transactional sex. Acceptance of wife-beating was measured using five questions specifying conditions when it is justifiable to beat a wife and probing if respondents endorse wife-beating under those situations. These questions demonstrated high internal consistency with an alpha score of 0.77. The scores were grouped into three, with zero indicating not endorsing wife-beating. A score of one indicated somewhat supports wife-beating, and a score of 2–5 indicated endorsement of wife-beating.

We considered four household-related factors: living with both parents, whether parents are alive or dead, parental support, and partner support (material and emotional provisioning). First, we asked if participants lived with their fathers and mothers and coded responses as living with one, both parents, or not. Similarly, we asked if their fathers and mothers were alive and coded responses as both parents alive, one parent dead, and both dead. Finally, participants rated the support they received from their parents, including material and financial support, as good, fair, poor, or no support. We also asked if participants belonged to a social group they met with regularly.

Three community-level factors were considered: place of residence, neighborhood safety nets, and safety. The place of residence was grouped as rural and urban. Neighborhood safety net was defined as having relationship resources to draw from in the community like friends, adult mentors, and other parents they could turn to if they had serious problems. Seven questions were used to measure safety nets, and all demonstrated high internal consistency (alpha coefficient 0.65). Higher scores indicate more community safety net. Lastly, neighborhood safety was measured using seven questions bordering on the feeling of safety walking around the community during the day and night, feeling scared of being raped, and being touched indecently, robbed, and teased in the past six months in the neighborhood. Higher scores indicate higher community safety.

### Statistical analysis

Analysis was performed using Stata 15. We ran descriptive statistics, including means, frequencies, and percentages, for all variables of interest. To answer the study objectives, we fitted multilevel mixed-effect logistics regression models. Given that the factors associated with IPV operate at multiple levels, we used a multilevel logistic regression analysis to estimate covariance at the individual/household and cluster/community level. Multilevel modeling adjusted the estimated standard errors, allowing for the clustering of observations within communities. This means respondents were nested within households and households nested within communities to account for cluster-level effects [34]. Model 1 was a null model with no covariates. We used this model to ascertain if the odds of experiencing IPV vary across randomly selected enumeration areas. Statistically significant intercept shows evidence that IPV exposure varies by EAs. In Model 2, we included individual-level factors such as age, marital status, employment, education, transactional sex, and endorsement of wife-beating.

Models 3 and 4 were used to examine the independent effects of household and community-level factors. Model 5 was a parsimonious model fitted to explore the main correlates of IPV among the PPAs. We used the ‘melogit’ command to fit the models. The log-likelihood ratio (LLR) and Akaike’s information criterion (AIC) tests were used to compare models with the highest log-likelihood and the lowest AIC indicating the best-fit model (see Table 3). Random effects were expressed in terms of community level variance, while the intra-class correlation coefficient (ICC) was used to examine clustering and the extent to which community/contextual factors explain the unexplained variance of the empty model [34]. All models were fitted at a 95% confidence level. P-values less than 0.05 were considered statistically significant.

## Results

### Descriptive findings

The mean age of the participants was 17.9 (SD 0.04) years. As shown in Table 1, most PPAs included in this study had no education/primary education (65.8%), never worked for pay (71.3%), resided in urban areas (62.1%), and did not endorse wife-beating (60%) or unequal gender norms (70.1%).

**Table 1:** Background characteristics of study participants by IPV experience

Variables	Never experienced IPV Freq (%)	Ever experienced IPV Freq (%)	All participants Freq (%)
Highest level of education			
No education/Primary	274 (67.6)	166 (62.1)	440 (65.8)
Secondary	129 (32.4)	100 (37.9)	229 (34.2)
Age			
13–16	41 (10.3)	47 (17.8)	87 (13.8)
17	78 (19.2)	55 (20.8)	133 (19.9)
18	122 (30.4)	77 (28.9)	199 (29.8)
19	162 (40.1)	87 (32.5)	249 (37.1)
Marital status			
Married	208 (51.5)	99 (37.2)	307 (45.8)
Separated	42 (10.4)	37 (14.0)	79 (11.8)
Single	153 (38.1)	130 (48.8)	283 (42.3)
Ever worked for pay			
Yes	106 (26.4)	85 (32.3)	191 (28.7)
No	297 (73.6)	181 (67.7)	478 (71.3)
Orphanhood status			
Double orphan	25 (6.2)	14 (5.1)	39 (5.8)
Single orphan	102 (25.3)	79 (29.8)	181 (27.1)
Non-orphan	276 (68.5)	173 (65.1)	449 (67.1)
Living with both parents			
Not living with both parents	214 (53.1)	127 (47.5)	341 (50.9)
Living with one parent	102 (21.3)	74 (27.8)	176 (26.3)
Living with both parents	87 (21.6)	65 (24.6)	152 (22.8)
Residence			
Rural	165 (38.0)	110 (38.4)	275 (38.2)
Urban	238 (62.0)	156 (61.6)	394 (61.8)
Parental support			
Good	271 (67.0)	154 (57.7)	425 (63.3)
Fair	70 (17.6)	66 (25.0)	136 (20.6)
Poor	21 (5.2)	12 (4.6)	33 (4.9)
No support	41 (10.3)	34 (12.7)	75 (11.2)
Partner support			
Good	277 (68.7)	138 (51.7)	415 (61.9)
Fair	51 (12.9)	57 (21.7)	108 (16.4)
Poor	21 (5.1)	27 (10.2)	48 (7.2)
No support	54 (13.3)	44 (16.5)	98 (14.5)
Endorse wife-beating			
Did not endorse	259 (64.5)	141 (53.2)	400 (60.0)
Somewhat endorses	73 (18.0)	41 (15.4)	114 (17.0)
Endorses	71 (17.5)	84 (31.4)	155 (23.0)
Ever engage in transaction sex			
Yes	38 (9.4)	49 (18.4)	87 (13.0)
No	365 (90.6)	217 (81.6)	582 (87.0)
Belong to social group			
No	257 (63.6)	55.3 (60.3)	404.0 (60.3)
Yes	146 (35.4)	119 (44.7)	265.0 (39.7)
Safety net in the community (mean)	403 (5.49)	266 (5.06)	669 (5.32)
Neighborhood safety	403 (3.99)	266 (4.23)	669 (4.08)

Note: Mean was computed for safety net in the community and neighborhood safety; we group 13 to16 years because only one participant was 13 years old, four were 14 years old and 20 were 15-year-old. Only one participant had no formal education and was coded with primary education

**Fig. 1:**
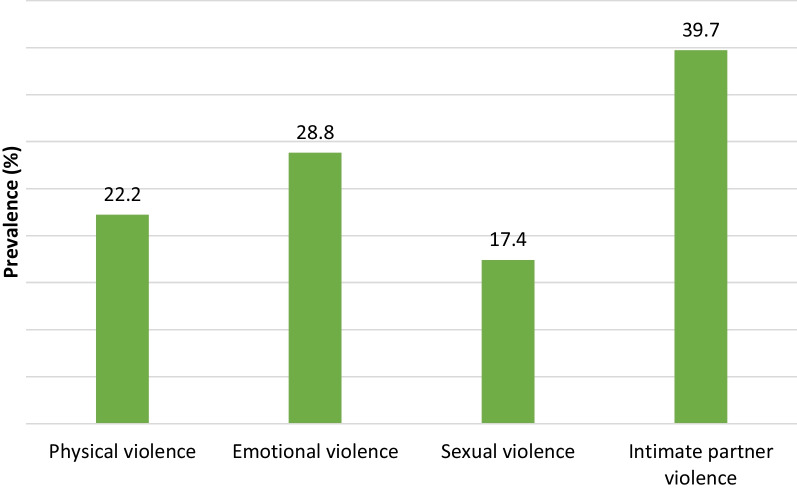
Prevalence of physical, emotional, sexual and initimate partner violence among pregnant and parenting adolescents

Figure 1 presents the prevalence of IPV among PPAs. While the prevalence of IPV was 39.7%, more PPAs had experienced emotional (28.8%) than physical (22.2%) and sexual (17.4%) violence. In addition, IPV prevalence was highest among girls who endorsed wife-beating (54.6%), engaged in transactional sex (56.4%), and received inadequate support from partners (56.7%).

We presented IPV prevalence by the individual-, household-, and community-level factors in Table 2. The prevalence of IPV was higher among girls aged 13 to 16 (53.3%), who were single (45.8%), engaged in transactional sex (56.4%), endorsed wife-beating (54.2%), and those who received poor (56.7%), fair (52.6%) or no support (45.0%) from their partners, compared to girls aged above 16, married, never engaged in transactional sex, did not endorse wife beating, and received good support from their partners.

**Table 2:** IPV prevalence by individual-, household-, and community-level factors

Variables	Never experienced IPV Freq (%)	Ever Experienced IPV Freq (%)
Age		
13–16	41 (46.7)	47 (53.3)
17	78 (58.4)	55 (41.6)
18	122 (61.5)	77 (38.5)
19	162 (65.2)	87 (34.8)
Marital status		
Married	208 (67.7)	99 (32.3)
Separated	42 (52.9)	37 (47.1)
Single	153 (54.2)	130 (45.8)
Ever worked for pay		
No	297 (62.3)	181 (37.7)
Yes	106 (55.3)	85 (44.7)
Highest level of education		
No education/Primary	274 (62.3)	166 (37.7)
Secondary	129 (56.4)	100 (43.6)
Ever engage in transaction sex		
No	356 (62.7)	217 (37.3)
Yes	38 (43.6)	49 (56.4)
Endorsement of wife beating		
Did not endorse wife beating	259 (64.8)	141 (35.2)
Somewhat endorse beating	73 (63.9)	41 (36.1)
Endorse beating	71 (45.8)	84 (54.2)
Orphanhood status		
Double orphan	25 (65.1)	14 (34.9)
Single orphan	102 (56.3)	79 (43.7)
Non-orphan	276 (61.4)	173 (38.6)
Living with both parents		
Not living with both parents	214 (62.9)	127 (37.1)
Living with one parent	102 (57.9)	74 (42.1)
Living with both parents	87 (57.1)	65 (42.9)
Belong to a social group		
No	257 (63.6)	147 (36.4)
Yes	146 (55.2)	119 (44.8)
Parental support		
Good	271 (63.7)	154 (36.3)
Fair	70 (51.6)	66 (48.4)
Poor	21 (63.0)	12 (37.0)
No support	41 (55.1)	34 (44.9)
Partner support		
Good	277 (66.8)	138 (33.2)
Fair	51 (47.4)	57 (52.6)
Poor	21 (43.3)	27 (56.7)
No support	54 (55.0)	44 (45.0)
Residence		
Rural	165 (60)	110 (40.0)
Urban	238 (60.4)	156 (39.6)
Safety net in the community (mean)	403 (5.49)	266 (5.06)
Neighborhood safety	403 (3.99)	266 (4.23)

Note: Mean was computed for safety net in the community and neighborhood safety. Only one participant had no formal education and was coded with primary education

### Multivariable findings

The results of the multilevel logistics regression models are presented in Table 3 below. Model 1 has no covariate and shows that IPV exposure significantly varies by clusters. In Model 2 (individual-level factors only), age 19 was significantly associated with lower odds of having ever experienced IPV, while single marital status, secondary education, endorsement of wife beating, and engaging in transaction sex were significantly associated with higher odds of having ever experienced IPV. In Model 3 (household-level factors only), only partner support was associated with IPV. Fair or poor partner support was associated with higher odds of IPV. In Model 4 (community-level factors only), only the perception of neighborhood safety was associated with IPV with a higher perception of neighborhood safety associated with lower odds of IPV. In Model 5, the association between age and IPV remained significant, although the strength of the association was weaker compared to Model 2. The association between marital status and IPV was no longer significant after fitting a parsimonious model, but the direction of effect persisted. Secondary education remained significantly associated with a higher risk of IPV in the adjusted model. Likewise, engaging in transactional sex continued to be significantly associated with higher odds of IPV, as was the endorsement of wife beating. A higher perception of neighborhood safety was significantly associated with lower odds of IPV, even after adjusting for individual and household factors.

**Table 3:** Multilevel logistics regression models showing individual-, household-, and community-level factors associated with IPV

Variables	Model 1	Model 2	Model 3	Model4	Model 5
Age					
13–16		1			1
17		0.63 (0.35–1.15)			0.69 (0.37–1.27)
18		0.59 (0.34–1.05)			0.65 (0.36–1.16)
19		0.45 (0.26–0.79)*			0.49 (0.27–0.87)*
Marital status					
Married		1			1
Separated		1.57 (0.90–2.74)			1.49 (0.79–2.80)
Single		1.57 (1.07–2.30)*			1.56 (0.95–2.55)
Ever worked for pay					
No		1			1
Yes		1.11 (0.75–1.65)			1.10 (0.73–1.66)
Highest level of education					
No education/Primary		1			1
Secondary		1.62 (1.11–2.36)*			1.72 (1.16–2.54)**
Ever engage in transaction sex					
No		1			1
Yes		2.28 (1.37–3.79)**			2.29 (1.35–3.89)**
Endorsement of wife beating					
Did not endorse wife beating		1			1
Somewhat endorse beating		0.87 (0.54–1.40)			0.80 (0.49–1.31)
Endorse beating		2.14 (1.39–3.31)***			1.97 (1.27–3.08)***
Orphanhood status					
Double orphan			1		1
Single orphan			1.27 (0.57–2.83)		1.93 (0.83–4.48)
Non-orphan			0.94 (0.43–2.03)		1.61 (0.71–3.64)
Living with both parents					
Not living with both parents			1		1
Living with one parent			1.21 (0.79–1.84)		0.87 (0.53–1.41)
Living with both parents			1.34 (0.83–2.14)		0.86 (0.49–1.49)
Belong to social group					
No			1		1
Yes			1.35 (0.95–1.91)		1.33 (0.92–1.91)
Parental support					
Good			1		1
Fair			1.38(0.89–2.13)		1.35 (0.86–2.14)
Poor			0.74 (0.33–1.66)		0.63 (0.27–1.48)
No support			1.65 (0.94–2.88)		1.62 (0.71.3.64)
Partner support					
Good			1		1
Fair			1.93 (1.20–3.10)**		1.66 (1.00–2.76)
Poor			2.50 (1.28–4.88)**		1.96 (0.97–3.96)
No support			1.52 (0.93–2.47)		1.07 (0.61–1.85)
Residence					
Rural				1	1
Urban				1.02 (0.65–1.59)	0.87 (0.56–1.36)
Safety net in the community				1.02(0.94–1.12)	1.04 (0.95–1.14)
Neighborhood safety				0.75 (0.65–0.87)***	0.81 (0.69–0.95)*
Intercept	0.62***	0.34*	0.34*	2.63	0.64
LR test vs. logistic model	15.15***	10.51***	9.39**	8.97**	4.36*
Log likelihood	− 442.01	− 418.00	− 428.23	− 433.87	− 403.85
ICC	0.105	0.093	0.085	0.081	0.060
AIC	888.01	860.00	882.46	877.73	859.69
BIC	897.02	914.07	941.04	900.26	976.84
Number of clusters	66	66	66	66	66

Exponentiated coefficients; 95% confidence intervals in brackets *p < 0.05, **p < 0.01, ***p < 0.001 Note: Only one participant had no formal education and was coded with primary education

## Discussion

Studies on the experience of IPV among PPAs in Africa are scarce. This gap in research could result in issues affecting pregnant and parenting girls being neglected in policies and programs. Our study addressed this gap by examining IPV prevalence and its risk and protective factors among PPAs in Malawi. Our analysis revealed that two in five PPAs had experienced IPV, indicating that IPV is common among this cohort and higher than the World Health Organization’s 24% global average among ever-partnered adolescent girls [1]. The prevalence of IPV in our study is also far higher than the 27% prevalence Kidman & Kohler [15] found among adolescents in rural Malawi and mirrors levels among all ever-married women in the 2015–16 Malawi Demographic and Health Survey [27]. Our findings are in line with the results of studies from the United States that show that adolescent mothers face a huge burden of IPV before, during, and after childbirth [19–23]. They also corroborate Tetteh et al.’s [35] findings demonstrating that teenage pregnancy is a risk factor for physical violence.

Our findings on the factors associated with IPV experience support the assumptions in the socio-ecological model. Factors influencing exposure to IPV operate at multiple levels, including at the individual, household, and community levels. At the individual level, our analysis shows that age, education, engaging in transactional sex, and acceptance of wife-beating are significantly associated with IPV. Surprisingly, increasing age was linked to lower odds of IPV. In contrast, Selin et al. [36] found a higher prevalence of sexual and physical IPV among older adolescents aged 17–20 compared to younger ones aged 13–16. However, our study population is a sub-category of adolescents with a higher risk of IPV [19–23]. One plausible explanation for our finding is sex with girls aged 13–16 is far more likely to be coerced than those aged 17 and older, given their limited ability to consent. This finding may not mean that IPV prevalence is generally higher among younger adolescent girls (aged 13–16) than older ones (above 16 years). But it reflects a disproportionate burden among younger PPAs, whose pregnancies most likely resulted from sexual violence.

Contrary to previous studies [37, 38], our study shows that PPAs with secondary education had higher odds of IPV than those with no education/primary education. Attaining an educational level of secondary education and above is expected to provide young women with skills and knowledge to buffer against IPV exposure. In most cases, education empowers women and raises their assertiveness, thereby minimizing IPV [39]. However, studies have also shown that the school environment is a risk factor for sexual violence [40, 41]. Therefore, it is plausible that men target school girls for sexual exploitation and adolescent girls with secondary education are likely victims. Adolescents with secondary education may also be more comfortable reporting IPV.

We also found that PPA girls who reported having engaged in transactional sex had higher exposure to IPV than those who never did. This finding is consistent with Alangea et al. in a study among women in the central region of Ghana [42]. We can draw some explanations from the literature exploring violence against sex workers to understand the link between transactional sex and IPV. First, while transactional sex differs from sex work, poverty is a critical facilitator of sex for money [43], and existing literature has shown that poverty is a risk factor for sexual violence [44]. Second, there is often a power imbalance in transactional relationships, with girls powerless because they depend on men or boys. Third, when girls engage in transactional sex, they usually have multiple partners [45]. Lack of loyalty to their partners could result in physical and emotional violence from these partners. In other words, their partners can physically and emotionally assault them as a punishment for not getting loyalty. Also, when men spend money on women, they often expect sex in return. Sexually entitled men are often controlling and could become violent if denied having spent on their women [46].

Consistent with previous research [47], our study shows that endorsement of wife-beating is associated with a higher risk of IPV. When girls accept wife-beating, they suffer from it and are more likely to remain in relationships detrimental to their overall well-being. They are also less likely to report it or seek help. This finding makes deconstructing the acceptance of wife-beating a significant priority for all interventions to end violence against women. The acceptance of wife-beating is rooted in the patriarchal systems of male dominance and superiority. Societal norms and values uphold patriarchy, thus increasing women and girls' risk of IPV [36]. The need to sustain marriage institutions also makes women endure various forms of IPV.

Our results also show that family structure in terms of living with parents or being an orphan did not increase PPA girls’ risk of IPV. Also, parental support in financial and material provision did not reduce the risk of IPV. However, partner support reduces the odds of IPV. The PPA girls who rated partner support as good had a lower likelihood of exposure to IPV. This finding suggests that supportive partners are less likely to be violent toward their partners. Support for partners is mainly in the form of provisions and emotional, and to a lesser extent, in completing household chores in the study setting.

Perceived neighborhood safety was the only community-level factor significantly associated with IPV. Rating the neighborhood as safe was significantly associated with lower odds of IPV. This finding is consistent with Popkin, Leventhal, and Weismann’s [48] study, which shows that IPV among adolescents is usually more prevalent in neighborhoods with high poverty levels and a wide range of social problems. Unsafe environments are characterized by aggravated gender inequality and gender-based violence. Unsafe neighborhoods may also have fewer law enforcement officers and be characterized by weak social ties and a low level of communal activities, which could increase the risk of IPV [49]. Perpetrators could easily escape punishment in this setting, thus, creating a culture of impunity.

### Policy and program implications

Our findings have significant implications for policies and programs in Malawi and other African countries. Given the massive burden of IPV among PPA girls, decision-makers need to prioritize this category of adolescents when developing policies and programs. Also, given the inextricable link between IPV and depression and poor health-seeking behavior for both, it is crucial to screen PPAs for experiences of IPV as part of maternal health care. Such screening would likely improve reporting and care-seeking.

### Strengths and limitations

This study is among the few studies on IPV among PPA girls in SSA. Our focus on the individual, household, and community-level factors associated with IPV is a significant strength of this study. However, there are some limitations, which we discuss to contextualize the findings. The cross-sectional nature of this study means causal relationships cannot be inferred between IPV and all the factors considered. The prevalence of IPV may also be underestimated because our study relied on self-reporting, which is prone to social desirability bias. Further, the generalizability of our findings is limited, given that our study took place only in Blantyre and not the entire country. Nonetheless, our results build on previous studies and could provide important information on IPV among PPAs lacking in the literature.

## Conclusion

Pregnant and parenting adolescents in Malawi are vulnerable to IPV, underscoring the need for appropriate interventions to address gender-based violence. Our findings highlight multiple individual, household, and community factors that may increase PPAs’ vulnerability to IPV. Interventions addressing IPV should target younger PPA (17 years or younger) and those who engage in transactional sex. In addition, interventions to change social norms that promote acceptance of violence are warranted as countries work towards achieving the Sustainable Development Goals (SGDs), which include specific targets for eliminating all forms of violence against women and girls.

## Data Availability

Data will be made available on reasonable request to the corresponding author.

## Abbreviations

EA: Enumeration area
IPV: Intimate partner violence
PPA: Pregnant and parenting adolescent
SDG: Sustainable Development Goal
